# Two new species of *Indigofera* L. (Leguminosae) from the Sneeuberg Centre of Floristic Endemism, Great Escarpment (Eastern and Western Cape, South Africa)

**DOI:** 10.3897/phytokeys.48.4798

**Published:** 2015-04-02

**Authors:** V. Ralph Clark, Brian D. Schrire, Nigel P. Barker

**Affiliations:** 1 Institut für Systematische Botanik, Zollikerstrasse 107, Universität Zürich, 8008 Zürich, Switzerland; 2 The Herbarium, Royal Botanical Gardens, Kew, Richmond, Surrey, TW9 3AB, United Kingdom; 3 Great Escarpment Biodiversity Programme, Department of Botany, Rhodes University, Grahamstown, 6140, South Africa

**Keywords:** Biodiversity, Cape Clade, Eastern Cape, endemic species, field exploration, Great Escarpment, *Indigofera*, Leguminosae, new species, Sneeuberg Centre, South Africa, Western Cape, taxonomy

## Abstract

Two new species of *Indigofera* L. (Leguminosae) are described from the Sneeuberg Centre of Floristic Endemism on the southern Great Escarpment, Eastern and Western Cape Provinces, South Africa. Both species are localised high-altitude endemics. *Indigofera magnifica* Schrire & V.R. Clark is confined to the summit plateau of the Toorberg–Koudeveldberg–Meelberg west of Graaff-Reinet, and complements other western Sneeuberg endemics such as *Erica passerinoides* (Bolus) E.G.H. Oliv. and *Faurea recondita* Rourke & V.R. Clark. *Indigofera asantasanensis* Schrire & V.R. Clark is confined to a small area east of Graaff-Reinet, and complements several other eastern Sneeuberg endemics such as *Euryops exsudans* B. Nord & V.R. Clark and *Euryops proteoides* B. Nord. & V.R. Clark. Based on morphology, both new species belong to the Cape Clade of *Indigofera*, supporting a biogeographical link between the Cape Floristic Region and the Sneeuberg, as well as with the rest of the eastern Great Escarpment.

## Introduction

The genus *Indigofera* L. (Leguminosae) is a large genus comprising some 750 taxa worldwide, with the majority (ca. 550) occurring in Africa and Madagascar (Schrire et al. 2009). In South Africa, *Indigofera* is represented in all biomes, but is particularly well represented in the Cape Floristic Region and the eastern Great Escarpment. The discovery of two new *Indigofera* species from the Sneeuberg Centre of Floristic Endemism (Clark et al. 2009) supports the view that continued field exploration is essential for biodiversity and biogeographical research in South Africa (Robertson and Barker 2006). This paper describes these two *Indigofera* species, complementing the descriptions of several other Sneeuberg discoveries since 2005 (Goldblatt and Manning 2007, Nordenstam et al. 2009, Stirton et al. 2011, Rourke et al. 2013).

## Materials and methods

The species were originally discovered in 2006 during intense plant collecting on the Sneeuberg as part of a plant diversity and biogeographical study of the southern Great Escarpment (Clark 2010, Clark et al. 2009). Extensive sampling from 2006–2012 in areas adjacent to the known populations of each species has not revealed other populations. Several return trips to the respective populations since their discoveries were necessary before suitable fruiting material could be obtained.

## Species treatments

### Indigofera magnifica

Taxon classificationPlantaeFabalesFabaceae

Schrire & V.R. Clark sp. nov.

urn:lsid:ipni.org:names:77146131-1

**Figure 1.**
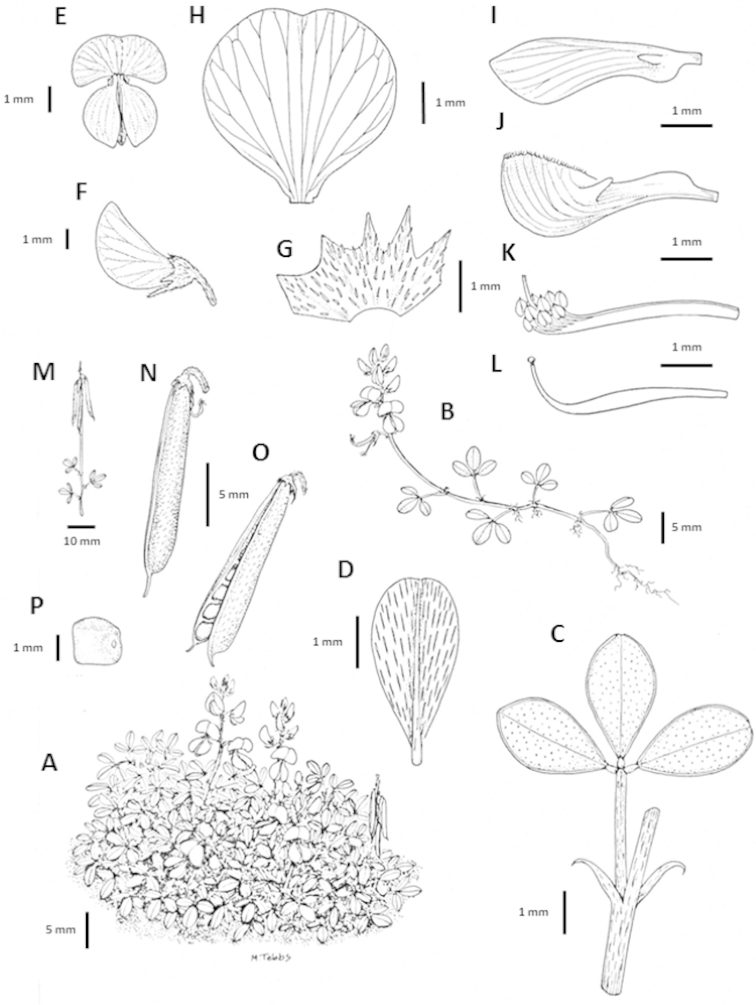
Analytical drawings of *Indigofera magnifica* Schrire & V.R. Clark, all drawn from the type collection (*Clark VR & Moholwa TT 206*) **A** growth habit **B** stoloniferous habit **C** trifoliolate leaf with stipules **D** terminal leaflet, underside **E** flower, front view **F** bud, side view **G** calyx **H** standard petal **I** wing petal **J** keel **K** staminal sheath **L** pistil **M** infructescence **N** pod **O** dehiscent pod **P** seed. Drawings by M. Tebbs.

**Plate 1.**
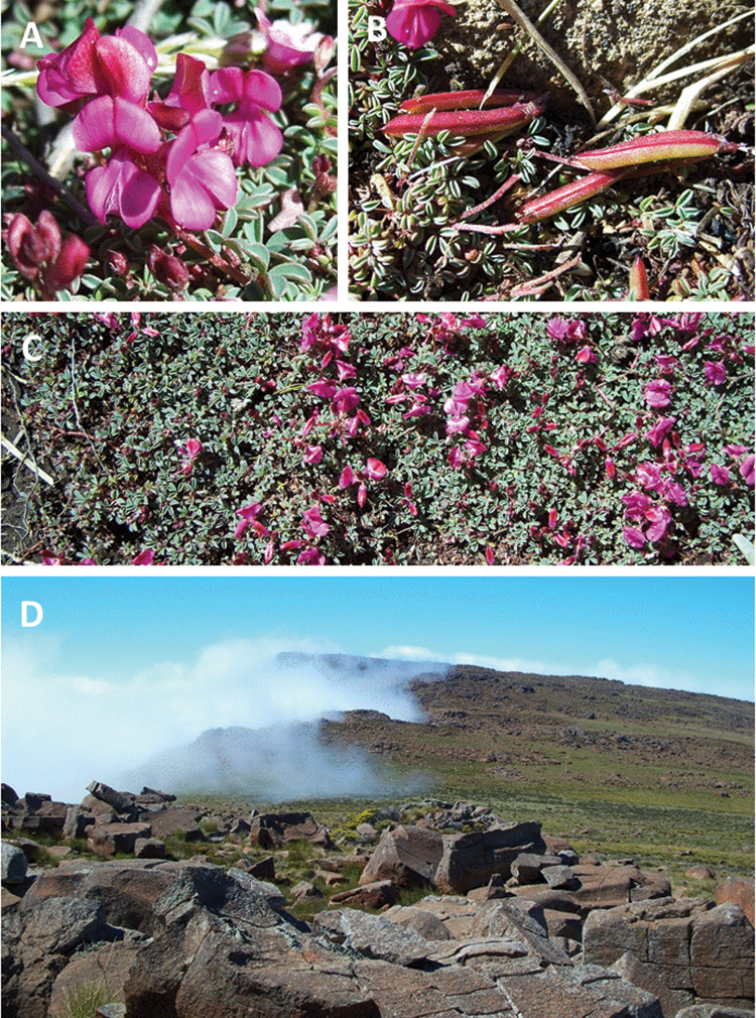
*Indigofera magnifica* Schrire & V.R. Clark, plants *in situ* on the Koudeveldberge, Sneeuberg (Western Cape Province; *Clark VR & Moholwa TT 206*) **A** inflorescence **B** fruits **C** typical prostrate growth habit **D** mountain summit habitat. Photographs by V.R. Clark.

**Figure 2.**
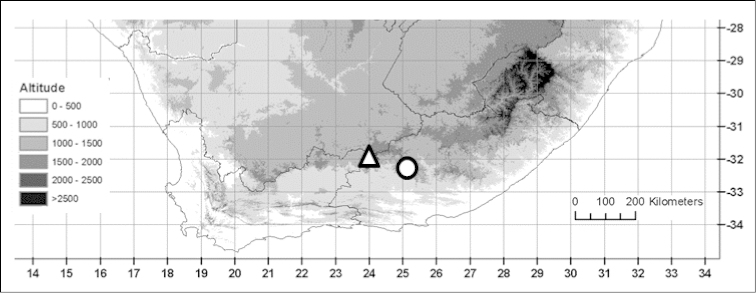
Known distributions of *Indigofera magnifica* Schrire & V.R. Clark (△) and *Indigofera asantasanensis* Schrire & V.R. Clark (○).

Figs 1 , 2 ; Plate 1

#### Diagnostic characters.

*Indigofera magnifica* is morphologically similar and most closely related to *Indigofera meyeriana* Eckl. & Zeyh., but differs in its prostrate, compact, densely matted habit (vs. laxly spreading, less dense and diffuse habit), sparsely to moderately strigose becoming reddish-maroon and glabrescent stems (vs. green-grey to canescent), fewer flowers (±3–8) per raceme (vs. >8), brighter, more vivid pink flowers (vs. paler pink), and sparsely, appressed hairy pods (vs. spreading hairy pods). The overall colour of plants of *Indigofera magnifica* is a darker green than the generally grey-green to grey appearance of *Indigofera meyeriana*. *Indigofera meyeriana* is common and widespread in the Western Cape and western Northern Cape Provinces, and also occurs on the Sneeuberg.

#### Type.

South Africa, Western Cape Province, 3224AA, Plaas 113: summit plateau of the Koudeveldberge, Murraysburg District, Sneeuberg. 32°10'32"S, 24°01'41"E, 2134 m, 9 December 2011, *Clark VR & Moholwa TT 206* (K, holotype; GRA, MO, NBG, NSW, PRE, S, isotypes).

#### Description.

*Prostrate suffrutex* 20–50 mm tall, densely to laxly matted, much branched. *Stems* slender, terete to ribbed, sparsely to moderately strigose with whitish biramous hairs, glabrescent later, reddish-maroon; stoloniferous, often rooting from nodes, arising from a woody rootstock. *Leaves* alternate, digitately trifoliolate, petiole 2–8 mm long, scattered with pearl bodies at base of leaflets. *Stipules* 1–2 mm long, up to 0.5 mm wide at base, lanceolate, attenuate, falcate, often recurved at apex, ± membranaceous, gland-tipped, reddish. *Stipels* absent. *Terminal leaflet* 1.5–5.5 mm × 1–3 mm, obovate, apex emarginate, truncate or rounded, base cuneate, upper surface glabrous or sparsely appressed strigose, paler than below; lower surface more densely strigose and slightly rugose; margins somewhat thickened, often appearing slightly involute, often reddish; lateral leaflets similar. *Racemes* (10)20–70 mm long, many times longer than the subtending leaf, including a peduncle of (6)11–55 mm, becoming flattened, appearing soft-tissued on drying; ±3–8 flowered; bracts 0.5–1.5 mm long, lanceolate-subulate, recurved at apex, caducous. *Pedicels* 0.75–1.5 mm long, becoming recurved in fruit. *Flowers* 4.5–6.5 mm long. *Corolla* vivid fuchsia-pink. *Calyx* 1.5–2.5 mm long, lobes triangular, 0.75–1.4 mm, ± equaling the tube, ± sparsely strigose appressed. *Standard* 5.5–6.5 mm long, up to 5 mm wide, broadly obovate, tapering to a short claw at the base; blade sharply reflexed upwards for distal half of length; apex rounded to emarginate; dorsal surface glabrous, often with translucent, short stripes. *Wings* 5–6 mm long, unguiculate, shortly clawed at base, asymmetrically obovate towards apex. *Keel petals* 5–6.5 mm long, valvately connate distally, lateral spurs to 1 mm long, distal margin curving upwards to base of the keel to an obtuse apex; claws ± 2 mm long, broadening from the base. *Stamens* 4–5 mm long, alternately long and short, the 9 fused stamens free for ± 1 mm distally; anthers uniform. *Ovary* densely strigilose laterally, glabrous along upper margin; stigma capitate. *Pods* (9)11–15 mm long, up to 3.5 mm wide, cylindrical, inflated, shiny, reddish-green becoming reddish-brown, sparsely strigose, explosively dehiscent with the valves twisting. *Seeds* 4–6, 1.5 × 1.5 mm, ± quadrate, dark green.

#### Etymology.

The specific epithet *magnifica* is derived from the Latin adjective *magnicus* –*a* –*um* (a. splendid, magnificent) and is named for the magnificent, showy, vivid fuchsia-pink flowers.

#### Distribution and ecology.

*Indigofera magnifica* is confined to the summit plateau of the Toorberg–Koudeveld–Meelberg in the western Sneeuberg, between 1700–2150 m. The species is occasional to abundant, found exclusively on the dolerite-derived loamy-clays and black turf soils typical of this plateau. The vegetation type is Karoo Escarpment Grassland (Gh1, Mucina and Rutherford 2006), typical of high altitudes in the Sneeuberg mountain complex, with the dominant grass species being *Tenaxia* (=*Merxmuellera*) *disticha* (Nees) N.P. Barker & H.P. Linder. *Indigofera magnifica* is particularly abundant on the highest parts of the plateau near the eastern and southern scarps, where it forms large colonies. It compliments a suite of several local endemics only found in the western Sneeuberg, including *Acmadenia* sp. nov., *Erica passerinoides* (Bolus) E.G.H. Oliv., *Euryops dentatus* B. Nord. and *Faurea recondita* Rourke & V.R. Clark (Clark et al. 2009, 2012, Rourke et al. 2013).

#### Conservation status.

While the extent of occurrence (EOO) of *Indigofera magnifica* is small (ca. 30 km^2^), it is common (probably >10 000 individuals) in its restricted area. There are no obvious risks from the current land-use of livestock grazing: plants do not show evidence of damage from foraging or trampling. The remote, rocky high-altitude habitat renders it relatively safe from other detrimental land-use. Already restricted to summit elevations, it is however potentially at risk from global climate change. Any potential wind farm proposals for the Toorberg–Koudeveld–Meelberg would place this species at serious risk. The category VULNERABLE (Vu D2) is thus recommended.

#### Further collections and localities.

South Africa, Western Cape Province (straddling the provincial boundary with the Eastern Cape Province), 3223BB & 3224AA, Farms Plaas 113, Annex Koudeveld 114, Koudvelds Hoogte 117, Annex Onder Hoogde 116: summit plateau of the Koudeveldberge and Meelberg, Graaff-Reinet and Murraysburg Districts, Sneeuberg. ca. 32°10–11'S 23°59'E, ca. 2100 m, 25 November 2006, *Clark VR & Te Water Naudé T 335* (GRA, K).

—Western Cape Province, 3224AA, Farm Quaggas Drift 108: Toorberg summit plateau, next to stream ca. 100 m from edge of escarpment, Murraysburg District, Sneeuberg. 32°08'46"S, 24°04'31"E, 1780 m, December 2007, *Clark VR & Pienaar C 511* (GRA, K).

### Indigofera asantasanensis

Taxon classificationPlantaeFabalesFabaceae

Schrire & V.R. Clark sp. nov.

urn:lsid:ipni.org:names:77146132-1

**Figure 3.**
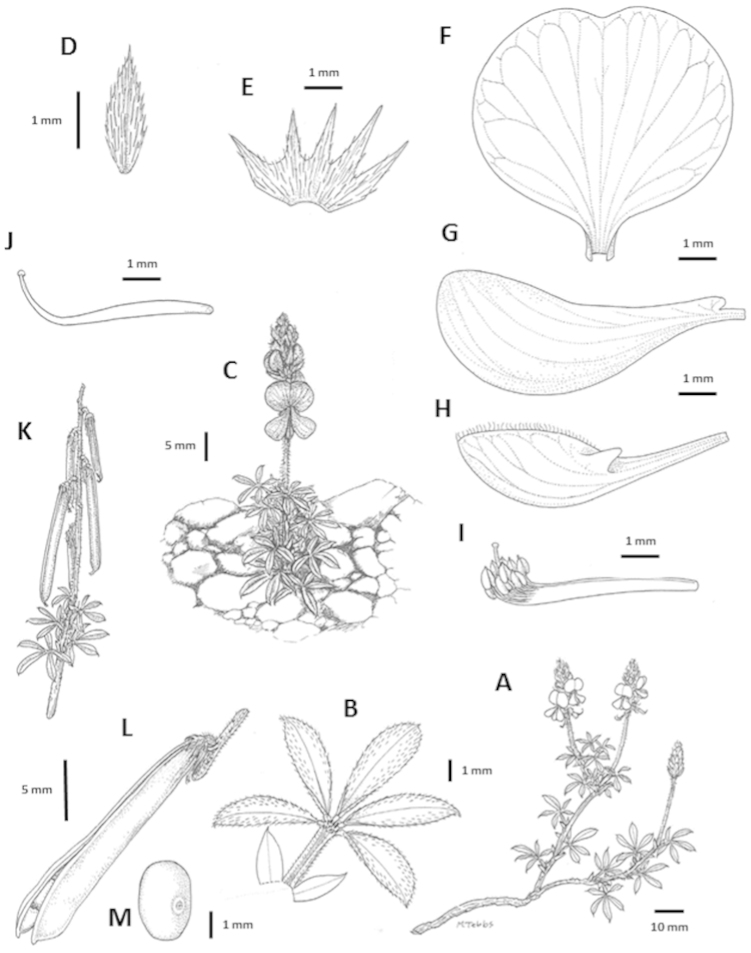
Analytical drawings of *Indigofera asantasanensis* Schrire & V.R. Clark, all drawn from the type collection (*Clark VR & Moholwa TT 211*) **A** growth habit **B** digitately foliolate leaf with stipules **C** growth habit **D** stipule **E** calyx **F** standard petal **G** wing petal **H** keel **I** staminal sheath **J** pistil **K** infructescence **L** dehiscent pod **M** seed. Drawings by M. Tebbs.

**Plate 2.**
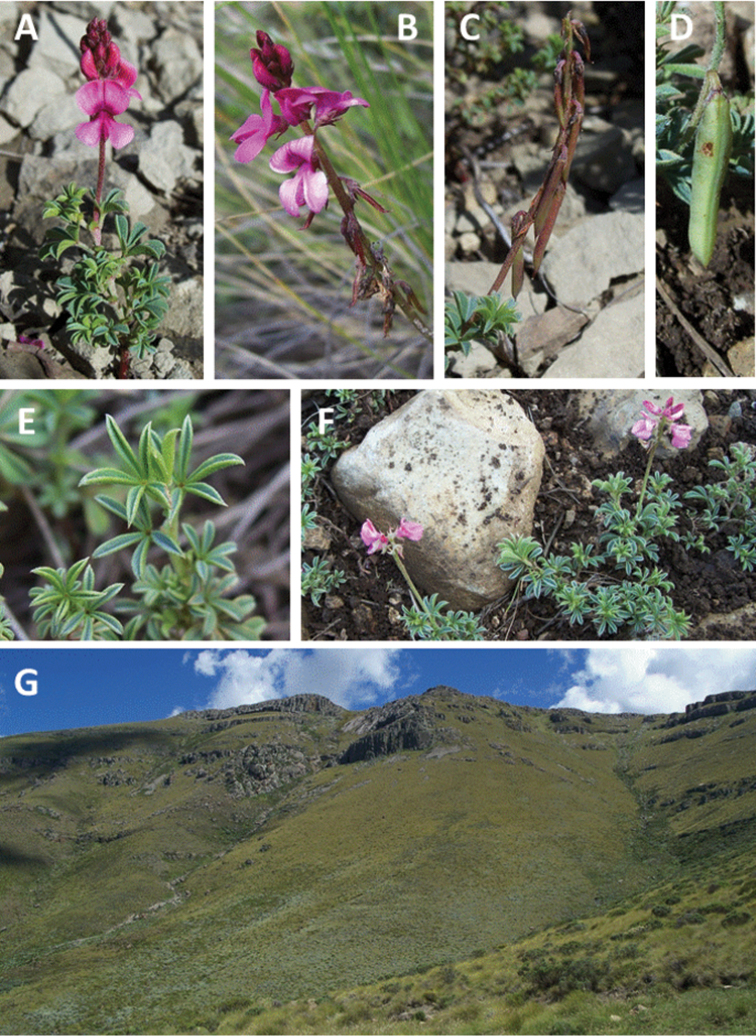
*Indigofera asantasanensis* Schrire & V.R. Clark, plants *in situ* on the slopes above ‘Suurkloof’, Asante Sana Private Game Reserve, Sneeuberg (Eastern Cape Province; *Clark VR & Moholwa TT 211*) **A** inflorescence from the front **B** inflorescence from the side **C** infructescence **D** close up of a single pod **E** the digitately foliolate leaves **F** growth habit **G** Escarpment mountain habitat. Photographs by V.R. Clark.

Figs 2 , 3 ; Plate 2

#### Diagnostic characters.

*Indigofera asantasanensis* is similar to *Indigofera alpina* Eckl. & Zeyh., but differs in its (3)5–7 foliolate leaves (vs. consistently trifoliolate leaves), leaflets 1.5–3 mm wide (vs. 4–16 mm wide), and stipules 1–1.6 mm wide (vs. 2–10 mm). *Indigofera asantasanensis* may also be confused with *Indigofera burchellii* DC., being similar in having digitately 5–7 foliolate leaves, but it has wider stipules (1–1.6 mm vs. < 0.5 mm). *Indigofera alpina* is mostly confined to the mountains of the Eastern Cape, while *Indigofera burchellii* is almost exclusively a southern Great Escarment species, centred from the Roggeveldberge to the Eastern Cape Drakensberg; both of these species also occur in the Sneeuberg.

#### Type.

South Africa, Eastern Cape Province, 3225AC, Farm 360: mountain slopes above Suurkloof, behind the old town of Petersburg, east of the Nardousberg, now included in Asante Sana Private Game Reserve, Graaff-Reinet District, Sneeuberg. 32°15'18"S, 25°00'10"E, 1708 m, 10 December 2011, *Clark VR & Moholwa TT 211* (K, holotype; GRA, MO, NBG, NSW, PRE, S, isotypes).

#### Description.

*Decumbent to erect suffrutex* 100–200 mm tall, much branched, densely leafy.

*Stems* slender, terete to strongly ribbed, angular, or longitudinally wrinkled, scattered with pearl bodies; moderately to densely strigose with spreading biramous hairs often crisped at the tips; reddish-brown, becoming woody below; a rhizomatous colony, diffusely branching from an indistinct woody rootstock. *Leaves* alternate, digitately (3)5–7-foliolate, petiole 1.5–5 mm long, deeply channelled above, scattered with pearl bodies at base of leaflets. *Stipules* 1.5–5 mm long, (0.75)1–1.6 mm wide, triangular to obliquely ovate-lanceolate, acuminate; amplexicaule, leaving annular sheath around stems, ± membranaceous. *Stipels* absent. *Terminal leaflet* (2.5)4–10 mm × (1)1.5–3 mm, obovate to oblanceolate, apex rounded, apiculate, often complicate; sparsely to moderately spreading or appressed strigose on both surfaces, hairs often coarser above than below, secondary venation ± prominent below; margins somewhat thickened, often appearing slightly involute; lateral leaflets similar. *Racemes* 25–120 mm long, many times longer than the subtending leaf, including a peduncle of 15–50 mm, moderately to densely strigose, scattered with pearl bodies; ± 12–35 flowered; bracts 3–4 mm long × ca. 1.5 mm, ovate-lanceolate, acuminate, caducous. *Pedicels* 0.5–2 mm long, reflexed. *Flowers* 6–7.5 mm long. *Corolla* deep pink, darker wine-red in bud. *Calyx* 2–3 mm long, lobes triangular to lanceolate, 1–2 mm long, ± equaling to twice as long as the tube, ± sparsely to densely strigose. *Standard* 5.5–6 mm × 5.5–6.5 mm, broadly obovate, tapering to a short claw at the base; blade sharply reflexed upwards for distal half of length; apex round to emarginate, dorsal surface glabrous, often with translucent, short stripes. *Wings* 5.5–6.5 mm long, unguiculate, shortly clawed at base, asymmetrically obovate towards apex. *Keel petals* 5–6 mm long, valvately connate distally, lateral spurs to 1 mm long, distal margin curving upwards to base of the keel to an obtuse apex; claws ± 2 mm long, broadening from the base. *Stamens* 4.5–5.5 mm long, alternately long and short, the 9 fused stamens free for ± 1 mm distally; anthers uniform. *Ovary* glabrous, stigma capitate. *Pods* 17–25 mm long, up to 3.5 mm wide, cylindrical, reddish-brown, glabrous, explosively dehiscent with the valves twisting. *Seeds* 4–5, 3 × 2 mm, subcylindrical, green.

#### Etymology.

The species is named for the Asante Sana Private Game Reserve, the owners and managers of which have been generous and instrumental in facilitating biodiversity research in the Sneeuberg. The known range of this species is almost entirely confined to this property.

#### Distribution and ecology.

*Indigofera asantasanensis* is currently only known from a small area in the eastern Sneeuberg from the Nardousberg to the Tandjiesberg–Coetszeesberg area behind the old town of Petersburg (now incorporated in the Asante Sana Private Game Reserve) and Pearston. The Tandjiesberg here is not to be confused with the more familiar Tandjiesberg (32°23'13"S, 24°42'13"E) of lower altitude and closer to Graaff-Reinet. *Indigofera asantasanensis* occurs from the mid-upper slopes to the summit plateau, ca. 1500–2200 m; it is locally abundant in Karoo Escarpment Grassland (Mucina and Rutherford 2006), dominated in this locality by *inter alia* *Tenaxia disticha*, *Euryops trilobus* Harv. and *Helichrysum splendidum* (Thunb.) Less. *Indigofera asantasanensis* occurs in loamy, rocky soils derived from both dolerite and Beaufort Group sandstone substrates. It compliments several local endemics, including *Euryops proteoides* B. Nord. & V.R. Clark and *Euryops exsudans* B. Nord. & V.R. Clark (Nordenstam et al. 2009).

#### Conservation status.

While the extent of occurrence (EOO) of *Indigofera asantasanensis* is small (ca. 15 km^2^), it is abundant (probably >10 000 individuals) in its restricted area. There are no obvious risks from the current land-use of game farming. The remote, rocky high-altitude habitat renders it relatively safe from other detrimental land-use. Already restricted to the higher elevations, it is potentially at risk from global climate change. Local infestations of the highly invasive grass *Nassella trichotoma* (Nees) Hack. ex Arechav. on Asante Sana Private Game Reserve and adjacent properties do constitute a potential risk to the habitat of *Indigofera asantasanensis*. The category Vulnerable (VU D2) is thus recommended.

#### Further collections and localities.

South Africa, Eastern Cape Province, 3225AC, Farm 360: mountain slopes above Suurkloof, mountains behind the old town of Petersburg, east of the Nardousberg, now in Asante Sana Private Game Reserve, Graaff-Reinet District, Sneeuberg. 32°15'17"S, 25°01'04"E, 1853 m, 6 December 2005, *Clark VR & Coombs G 208* (GRA, K).

—Farms Paardekom 5 and Annex Waterkloof 2: upper mountain slopes (Tandjiesberg-Coetszeesberg) ca. 15 km east of the Nardousberg (Sneeuberg), behind Pearston, Graaff-Reinet District. 32°16'47"S, 25°05'25"E, ca. 1950 m, 13 December 2006, *Clark VR & Coombs G 635* (GRA).

—Farm 360: mountain slopes above Suurkloof, mountains behind the old town of Petersburg, east of the Nardousberg (Sneeuberg), now in Asante Sana Private Game Reserve, Graaff-Reinet District 32°15'40"S, 25°10'10"E, 1550–2000 m, 31 March 2008, *Clark VR & Crause C 2* (GRA).

—3224BB, Upper Waterkloof 352: eastern end of Nardousberg ridge-line (Sneeuberg), Asante Sana Private Game Reserve, Graaff-Reinet District. 32°14'48"S, 24°55'58"E, 2171 m, 2 April 2008, *Clark VR & Crause C 34* & *65* (GRA).

## Biogeography

Including these two described species, some 20 of the Sneeuberg’s ca. 26 endemic species are concentrated in the moister south-western and south-eastern scarp areas. Sixteen species are local to one or the other of these two areas, the other four species with populations separated by the internal ‘Sunday’s River Interval’ (Clark et al. 2009, Stirton et al. 2011). Only one endemic (Hilliard and Burtt’s 1985, *Conium* sp. no. 4) occurs throughout the Sneeuberg, and a further three can be considered arid-adapted. This suggests that endemism in the Sneeuberg is driven by the availability of moisture, or that the moister scarp slopes and adjacent summits are climatic refugia for previously more widespread species (Clark and Barker 2014). This mirrors the patterns evident in the Main Drakensberg (Pooley 2003) and the Great Winterberg–Amatholes (Clark et al. 2014), where endemism is also highest on the wettest scarps and summits. *Indigofera magnifica* and *Indigofera asantasanensis* show extreme versions of this localism, being confined to unusually small areas on the summit in the west (*Indigofera magnifica*) and on the moist scarp slope in the east (*Indigofera asantasanensis*). Both are also located in the Cape Clade of *Indigofera* (Schrire et al. 2009), supporting a biogeographical connection between the Sneeuberg and the Cape Floristic Region, as well as with the rest of the eastern Great Escarpment.

## Supplementary Material

XML Treatment for Indigofera magnifica

XML Treatment for Indigofera asantasanensis
